# Combining MALDI-TOF and genomics in the study of methicillin resistant and multidrug resistant *Staphylococcus pseudintermedius* in New Zealand

**DOI:** 10.1038/s41598-018-37503-9

**Published:** 2019-02-04

**Authors:** Shahista Nisa, Clément Bercker, Anne C. Midwinter, Ian Bruce, Chris F. Graham, Pierre Venter, Allan Bell, Nigel P. French, Jackie Benschop, Karen M. Bailey, David A. Wilkinson

**Affiliations:** 1 Molecular Epidemiology and Public Health Laboratory, Hopkirk Research Institute, Massey University, Palmerston North, New Zealand; 2 Ecole Nationale Veterinaire de Toulouse, Toulouse, France; 3 NZVP (IDEXX), Palmerston North, New Zealand; 4 Gribbles Veterinary, Christchurch, New Zealand; 5 Fonterra Research & Development Centre, Palmerston North, New Zealand; 6 Dermvetonline, Auckland, New Zealand; 7 New Zealand Food Safety Science and Research Centre, Massey University, Palmerston North, New Zealand

## Abstract

*Staphylococcus pseudintermedius* is an opportunistic and emerging zoonotic pathogen that primarily colonises the skin of dogs. Many common variants are methicillin resistant (MRSP) or multidrug resistant (MDR), and drug resistance is increasingly reported across the globe. In New Zealand, MRSP isolation remains rare in clinics. To pre-emptively inform diagnostic and antimicrobial stewardship practices, we examine isolates of *S*. *pseudintermedius*, MRSP and MDR-MRSP from New Zealand dogs using a combination of methodologies. Genetic and genomic data combined with antimicrobial susceptibility screening identify common drug-resistance profiles and their genetic determinants. We demonstrate that sensitive and specific species-level identification of *S*. *pseudintermedius* can be achieved using Bruker MALDI-TOF MS and, further, that this technique can be used to identify some common subtype variants, providing a level of categorical precision that falls somewhere between single-locus and multi-locus sequence typing. Comparative genomics analysis of global *S*. *pseudintermedius* data shows that MRSP moves frequently across the globe, but that horizontal gene transfer events resulting in the acquisition of the SCC*mec* cassette (responsible for beta-lactam antibiotic resistance) are infrequent. This suggests that biosecurity and surveillance in addition to antibiotic stewardship should play important roles in mitigating the risk of MRSP, especially in countries such as New Zealand where MRSP is still rare.

## Introduction

Staphylococci are ubiquitous gram-positive bacteria, of which many are opportunistic pathogens that commonly constitute part of the natural skin flora of diverse animal species^1,2^. In humans, the main infecting species is *Staphylococcus aureus*, while in dogs, and to a lesser extent cats, *Staphylococcus pseudintermedius* has been shown to be the most common skin-associated coagulase-positive staphylococcal species^2^. Taxonomically, *S*. *pseudintermedius* belongs to the Staphylococcus Intermedius Group (SIG), which includes two other coagulase-positive species; *Staphylococcus delphini* and *Staphylococcus intermedius*^3^. These species share many phenotypic characteristics, and *S*. *pseudintermedius* was only recognised as a novel species in 2005, previous to which it was referred to as *Staphylococcus intermedius*^4^. *S*. *pseudintermedius* is also an emerging zoonotic pathogen and cases of *S*. *intermedius* in humans existed before the reclassification of *S*. *pseudintermedius*^5–8^.

As in many bacteria, antibiotic resistance is an increasing problem in *Staphylococcus* infections leading to major challenges to combat disease in both human and veterinary medicine^9^. Methicillin-resistant *S*. *pseudintermedius* (MRSP) and multidrug-resistant *S*. *pseudintermedius* including methicillin-susceptible and resistant variants (MDR-MSSP and MDR-MRSP) are found worldwide^10^. MRSP is of particular concern because beta-lactam antibiotics are often the first choice of treatment for *Staphylococcus*-associated infections^11^. While there are no known differences in virulence or invasiveness between MSSP and MRSP strains^12^, favourable clinical outcomes from MRSP infection largely rely on the timely identification of antimicrobial susceptibilities followed by suitable antibiotic treatment^13^. However, many of the predominant lineages of MRSP, such as members of clonal complexes (CCs) 71 and 45, have resistance to as many as 7 different classes of antibiotic and their prevalence is reported to be increasing in multiple geographic settings^10^. In veterinary medicine, where the use of important human antimicrobials such as vancomycin, daptomycin and linezolid is increasingly discouraged (eg.^14^), this effectively renders some of these lineages extensively, or pan-drug resistant (XDR, PDR) to their recommended and available antibiotics. These variants are commonly carried on the skin and oral mucus membranes of healthy animals, and no effective strategy exists for the removal of commensal association^13^ leaving surveillance and monitoring as the only effective approaches to reduce the burden of antimicrobial resistance^15^.

MRSP was first identified in New Zealand in 2014^16,17^ and it remains an uncommon laboratory isolate. Routine diagnostic methods currently used for identification of *S*. *pseudintermedius* and MRSP are relatively slow. The current diagnostic procedure for *S*. *pseudintermedius* differs between veterinary microbiology laboratories, and involves either the use of hyaluronidase and DNase biochemical tests or an initial test of polymyxin B susceptibility. Members of the SIG are most often hyaluronidase negative, DNAse positive and susceptible to polymyxin B, whereas *S*. *aureus* is intrinsically resistant to polymyxin B^18^ and demonstrates hyaluronidase activity^19^. Presumed *S*. *aureus* are tested for cefoxitin susceptibility and reported as either *S*. *aureus* or methicillin-resistant *S*. *aureus* (MRSA). Presumed members of the SIG are tested for oxacillin susceptibility and are reported as either SIG or methicillin-resistant SIG, however this classification may include other similar coagulase positive staphylococci such as *Staphylococcus schleiferi*. The use of matrix-assisted laser desorption/ionization time-of-flight mass spectrometry (MALDI-TOF MS) for pathogen identification is increasing, but there are varying reports of its specificity for members of the SIG^20–22^. Antimicrobial susceptibility testing of isolates by standard methods still takes several hours of incubation (commonly overnight).

The objectives of this study were to validate a rapid diagnostic test for *S*. *pseudintermedius* for application in New Zealand veterinary laboratories and to provide additional information surrounding antimicrobial resistance in New Zealand MRSP in order to inform antibiotic stewardship practices. We tested the discriminatory power of MALDI-TOF MS and its utility in differentiating variants of *S*. *pseudintermedius* found in New Zealand. Additionally, we used whole genome sequencing (WGS) and comparative genomics to characterise the resistance profiles, diversity and origins of New Zealand *S*. *pseudintermedius* strains. We show that MALDI-TOF MS accurately identifies *S*. *pseudintermedius* and some of its more common variants. Additionally, we find that MRSP is likely to have been imported into New Zealand on numerous occasions and provide genetic evidence supporting autochthonous transmission of some MRSP lineages. Using WGS-derived phylogenies we demonstrate that acquisition of the SCC*mec* cassette by horizontal gene transfer has occurred less frequently than has previously been estimated and that this difference in estimation is due to limitations in the phylogenetic resolution provided by seven gene MLST data.

## Materials and Methods

### Sample collection and isolation

In response to earlier observations of MRSP infection in dogs in New Zealand^16,17^, laboratory cultures of coagulase positive *Staphylococcus* spp. opportunistically collected from routine practice cases were supplied to ^*m*^EpiLab, Massey University by Gribbles and NZVP-IDEXX veterinary laboratories. Isolates came from across New Zealand between December 2015 and May 2017, and included the earliest isolate originating from the index study in 2014^16^; 117 samples in total, of which 106 were from dogs and 11 from cats. Where possible, two independent colonies were isolated from each sample on Tryptic Soy Agar (TSA) plates at 37 °C to ensure clonality, resulting in 229 separate isolates.

### MALDI-TOF

Isolate identification was carried out using full protein extraction (FPE) in the Bruker Biotyper MALDI-TOF. Ethanolic suspensions of overnight TSA culture were generated by homogenisation of approximately 2 mm^3^ of bacterial material in 300 µL of sterile high grade water, followed by addition of 900 µL of high grade 100% ethanol. Targets were prepared following the manufacturer’s recommendations. MALDI-TOF spectra, predictions of species, and log-score estimates of certainty were obtained in triplicate for each isolate using Bruker’s software and version 7 of Bruker’s isolate database (7311 RUO).

### Antimicrobial resistance screening

All isolates were assessed for their susceptibility to a panel of antimicrobials. The panel included oxacillin, amoxycillin-clavulanate (Australian approved name), trimethoprim, doxycycline, enrofloxacin, clindamycin, ciprofloxacin, chloramphenicol, cefpodoxime, cephalexin, cefovecin, amoxycillin (Australian approved name), novobiocin, oxytetracycline, gentamicin and ampicillin using the standard CLSI disk diffusion protocol, and validation control strain ATCC 25923 (ESR 917).

Resistant, intermediate and susceptible classes of resistance were assigned for each antibiotic referring to CLSI standards from CLSI VET08, Edition 4 or CLSI M100, Edition 27 where appropriate. Inducible clindamycin resistance was assessed by disk diffusion using the D-zone test as described by^23^.

In addition, we assessed the ability of the disk diffusion assay to predict the resistance genotype of each isolate from our data set where gene presence/absence data were available (either from genomic or PCR data, see below). Zone diameter upper and lower bounds were calculated that maximised the positive predictive value of phenotype (diameter) for genotype (presence/absence).

### Molecular Biology

Crude DNA extracts were prepared by boiling approximately 2 mm^3^ of bacterial material for 10 minutes in 1 mL of 2% Chelex™ (BioRad) suspension. The species of each isolate was verified using PCR primers and protocols for *Staphylococcus aureus*, *Staphylococcus intermedius* and *Staphylococcus pseudintermedius* described by Sasaki *et al*.^24^. PCR conditions were optimised using positive control strains ATCC 25923 (*S*. *aureus*), ATCC 29663 (*S*. *intermedius* Hajek) and in-house *S*. *pseudintermedius* controls that had been characterised by whole genome sequencing as well as negative control strains PNAL 93/21525 (*S*. *felis*), 87/1493 (*S*. *schleiferi*) and ATCC l2228 (*S*. *epidermidis*). Presence/absence of the *mecA* gene was screened in each isolate using the protocol of Lee *et al*.^25^, which was validated using in-house positive and negative control strains for which both phenotypic and genomic data were available.

Purified genomic DNA was extracted using a QIAamp Mini kit (Qiagen) following the manufacturer’s instructions with minor modifications. Genomic DNA was prepared for sequencing using the Nextera XT library kit (Illumina, San Diego, USA). Libraries were sequenced on an Illumina HiSeq using 2 × 250 bp paired end sequencing by the Massey Genome Service (New Zealand).

### Bioinformatics

Illumina reads were trimmed using a combination of in-house software and Trimmomatic^26^ and assembled using SPAdes version 3.9.0^27^ as part of the Nullarbor^28^ pipeline (https://github.com/tseemann/nullarbor). Nullarbor and seqkit^29^ were also used to extract the assembly statistics (Table S1). Assembled draft genomes were deposited to NCBI under BioProject number PRJNA473042.

Seven-gene MLST alignments and isolate data were obtained for all isolates recorded in PubMLST^30^ using the mlst software package (https://github.com/tseemann/mlst). Phylogenetic analysis of concatenated MLST sequences was performed in BEAST2^31^ with an MCMC chain length of 500,000,000 using an HKY substitution model accounting for invariant sites, a strict molecular clock and a Yule population size model. Convergence of the Bayesian phylogenetic inference was verified in Tracer v1.6 (http://beast.bio.ed.ac.uk/Tracer), with all estimated parameters returning estimated sample sizes values greater than 200 after a 10% burn-in.

For comparative genomic analyses, an extensive literature survey was performed at the start of March 2018 to identify potential sources of *Staphylococcus pseudintermedius* genomes, which were subsequently downloaded from ENA, NCBI and SRA databases or supplied directly from the authors of these published works. A total of 248 genomes were obtained for comparative analyses, including the 29 genomes generated in this study. SRA datasets were assembled using the methodology presented above. Clusters of orthologous groups were defined using the R package ‘pewit’ (https://github.com/iferres/pewit)^32^. Preliminary analyses showed that many published assembled genomes were highly fragmented, leading to errors in gene prediction and significant increase in per-genome gene number and a reduction in the number of identifiable core genes. Therefore, genomes were excluded from our comparative analysis based on the N50 statistic until all 7-gene MLST loci were identified as core genes in our clustering pipeline. The final threshold was an N50 value higher than 20,000. This resulted in 223 genomes being included in the final analysis. Gubbins^33^ was used to trim predicted mutation hotspot regions from the concatenated core gene alignment, which was then used for whole genome sequence (WGS) phylogeny. WGS phylogeny was performed using BEAST2^31^, with an MCMC chain length of 2 * 10^9^ using an HKY substitution model accounting for invariant sites, a strict molecular clock and a Yule population size model. As above, all estimated parameters returned estimated sample size values greater than 200 after a 50% burn-in. Time to most recent common ancestor estimates were performed in a similar way, integrating tip dates into the analysis and optimising the parameter posteriors for both strict and relaxed clock models over 10^8^ MCMC chains. Ancestral state reconstructions were performed from the output trees of the BEAST analysis in Mesquite^34^, defining either geographical origin or SCC*mec* variant as a categorical variable associated with each tip. Ancestral states were inferred using a parsimony model and the average number of estimated state changes was calculated over all trees. Network analysis was performed in the ‘igraph’ R package^35^, community analysis of geographic exchanges was performed using fast greedy clustering after summing directional measures to generate an undirected graph.

Presence/absence of antibiotic resistance genes was assessed using abricate (https://github.com/tseemann/abricate) and the ResFinder database^36^. Resistance associated point mutations at *gyrA* and *glrA* loci were identified by examining alignments generated using the Geneious R10 software package^37^.

The SCC*mec* type of each MRSP isolate was assigned based on its *ccrA* and *ccrB* gene composition using abricate (as above) and relevant reference sequences as specified by http://www.staphylococcus.net/Pages/SCCmecTypingEN.html and http://www.sccmec.org. Unambiguous matches of >95% identity were considered sufficient to attribute the presence of a *ccr* gene type. Ambiguous or inconclusive matches were curated manually using the Geneious R10 software package^37^. Data manipulation and visualisation was performed using the R packages ‘ggtree’^38^ and ‘ggmap’^39^.

MALDI-TOF spectral data analysis was performed using the ‘MALDIquant’ R package^40^. Peaks were aligned using the Lowess warping function at a tolerance of 0.01, and binned at a tolerance of 0.002. Linear discriminant analysis and peptide profiling were performed using the ‘sda’ R package^41^ and discriminant performance of peak combinations was assessed using the ‘crossval’ R package^42^.

## Results

### MALDI-TOF accurately identifies New Zealand *S*. *pseudintermedius* isolates

This study included 229 coagulase positive *Staphylococcus* spp. isolates obtained from veterinary pathology laboratories around New Zealand. MALDI-TOF MS was used for species-level identification of all isolates and the accuracy of these results was evaluated using PCR with primers specific for *S*. *aureus*, *S*. *intermedius* and *S*. *pseudintermedius*^24^. Isolates were also tested for novobiocin, polymyxin B and acriflavine sensitivities which have been shown to discriminate between coagulase positive/negative staphylococci or between *S*. *aureus* and members of the SIG^43^.

A total of 174 isolates were confirmed as *S*. *pseudintermedius* by PCR. MALDI-TOF MS identified 176 isolates as *S*. *pseudintermedius* with a median MALDI-TOF log-score of 2.33 (IQR: 2.23–2.42), corresponding to a sensitivity of 0.99 and a specificity of 0.93 when setting the PCR result as the gold standard. Five *S*. *pseudintermedius* isolates confirmed by MALDI-TOF MS and PCR were also positive with *S*. *aureus* PCR, suggesting a specificity of the *S*. *aureus* PCR of less than 100%. None of the isolates collected were identified as *S*. *intermedius* by either MALDI-TOF MS or PCR. We found that polymyxin B, acriflavine and novobiocin sensitivity cut-offs were all imperfect methods for the identification of *S*. *pseudintermedius* (Fig. S1).

### Beta-lactam antibiotic susceptibility and its determinants in New Zealand *S*. *pseudintermedius*

Beta-lactam resistance is conferred by the *mecA* or, less commonly, *mecC* genes that encode a penicillin-binding-protein (PBP2a) carried on a mobile chromosomal cassette called SCC*mec*^44^. MecA confers broad-spectrum beta-lactam resistance to many commonly used antibiotics. We assigned methicillin resistance or susceptibility based on the result of the CLSI disk diffusion protocol to oxacillin, and identified the presence or absence of the *mecA* gene by PCR for each isolate. Sixty-seven of the 176 presumptive *S*. *pseudintermedius* isolates (38%) were *mecA* positive. All oxacillin resistant isolates but one were identified as carrying the *mecA* gene.

We correlated the *mecA* presence/absence genotype with the results of the disk diffusion assay for other beta-lactam antibiotics, assigning isolates as susceptible, intermediate or resistant where CLSI breakpoints were available. Within our dataset, cephalexin, cefpodoxime and cefovecin zone diameters were all reliable predictors of the presence of the *mecA* gene (Table 1) with clear, well-separated upper and lower diameter boundaries that separated *mecA*^+^ and *mecA*^−^ isolates. Numerous isolates with intermediate resistance to cefpodoxime and cefovecin and a single isolate classified as susceptible to cefovecin possessed the *mecA* gene.

**Table 1:** Optimal upper and lower bounds of zone diameter for predicting antibiotic resistance genotype from disk diffusion phenotype in our dataset.

Antibiotic	Lower Bound (mm)	Upper Bound (mm)	Positive Predictive Value for genotype^*^	Resistance genotype
Oxacillin	16.5	22.5	0.98	*mec*A+
Ampicillin	17.5	17.5	0.9	*mecA*+
Amoxycillin	20.5	20.5	0.97	*mecA*+
Amoxycillin + clavulanate	28.5	28.5	0.82	*mecA*+
Cephalexin	22.5	26.5	0.97	*mecA*+
Cefpodoxime	20.5	25.5	0.98	*mecA*+
Cefovecin	25.5	29.5	0.97	*mecA*+
Oxytetracycline	11.5	27	1 (S = 1, n = 12)	*tetM*+
Doxycycline	17.5	28	1 (S = 1, n = 12)	*tetM*+
Ciprofloxacin	11.5	28.5	1 (S = 1, n = 16)	GyrA 84 L and Glr 80I
Enrofloxacin	12.5	25	1 (S = 1, n = 16)	GyrA 84 L and Glr 80I
Trimethoprim	5.5	19.5	1 (S = 1, n = 19)	*dfrG*+
Clindamycin	19	25	1 (S = 0.94, n = 18)	*ermB*+
Chloramphenicol	10.5	23.5	1 (S = 1, n = 15)	cat(pC221)+
Gentamicin	18.5	23	1 (S = 1, n = 18)	*aac(6)/aph(2)* + **

^*^For non-beta-lactam class antibiotics, positive predictive values are calculated for the 29 isolates which had genomes sequenced as part of this study. The sensitivity (S) of the test and the number (n) of resistance gene-containing genomes is shown in parentheses.

**Gentamicin resistance is also associated with genes *aph*III and *ant*IV in the sequenced NZ isolates.

**Figure 1:**
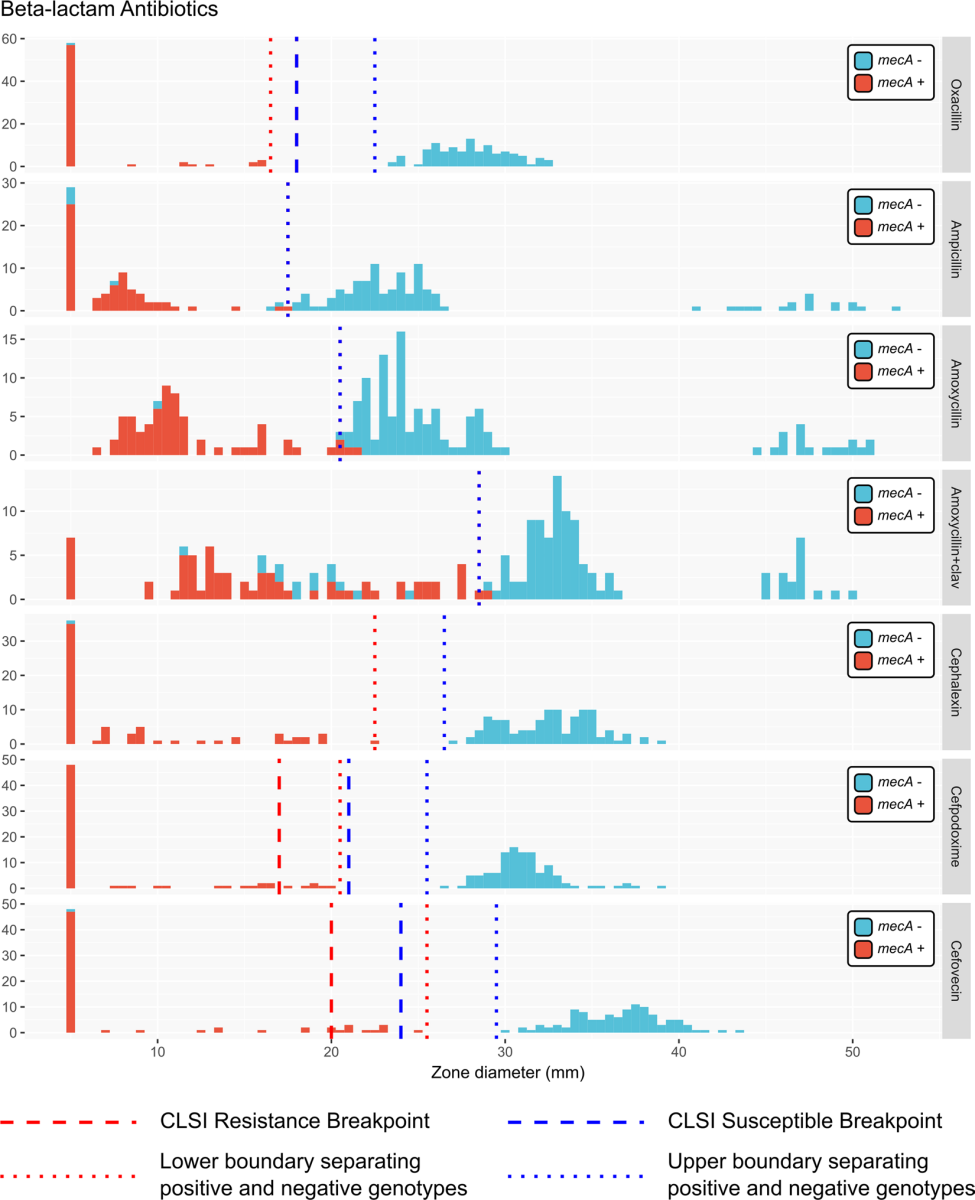
Beta-lactam antibiotic zone diameter measurements for all *S*. *pseudintermedius* isolates in this study. Histograms are presented for each antibiotic. A zone diameter of 5 mm is used to indicate a “no zone” result. Available genetic data are shown by the colour of each histogram, where antibiotic resistance gene positive genotypes are coloured light red, and negative genotypes are coloured light blue. Individual legends specify the relevant antibiotic resistance gene for each plot. Dotted lines specify the calculated optimal bounds for separating positive and negative genotypes in our data. Dashed lines show the R/I/S CLSI boundaries for antibiotics where breakpoint data were available at the time of publication.

Many isolates showed no sensitivity to the ampicillin disk but lacked the *mecA* gene. Ampicillin resistance may be conferred by a variety of other beta-lactamase encoding (*bla*) genes in staphylococci^45^. A subset of isolates lacking the *mecA* gene showed increased resistance to amoxycillin/clavulanate. To our knowledge there is no known specific mechanism for amoxycillin/clavulanate resistance, and interestingly all *mecA*^−^ isolates with increased amoxycillin/clavulanate resistance showed similar susceptibilities to amoxicillin as all other *mecA*^−^ isolates. A second subset of oxacillin sensitive isolates were also hypersensitive (showed approximately 100% increased zone diameters) to ampicillin, amoxycillin and amoxycillin/clavulanate (Fig. 1).

### Whole genome sequencing identifies New Zealand MRSP diversity and resistance genotypes

MDR variants of *S*. *pseudintermedius*, including MDR-MRSP, have been associated with specific genetic lineages or clonal complexes^10^. In order to examine the diversity of drug resistant variants obtained in New Zealand, we conducted sensitivity measurements for eight additional antibiotics from six antimicrobial classes commonly used to describe *S*. *aureus* resistance profiles^46^, and assigned susceptible, intermediate and resistant profiles where CLSI breakpoints were available.

**Figure 2:**
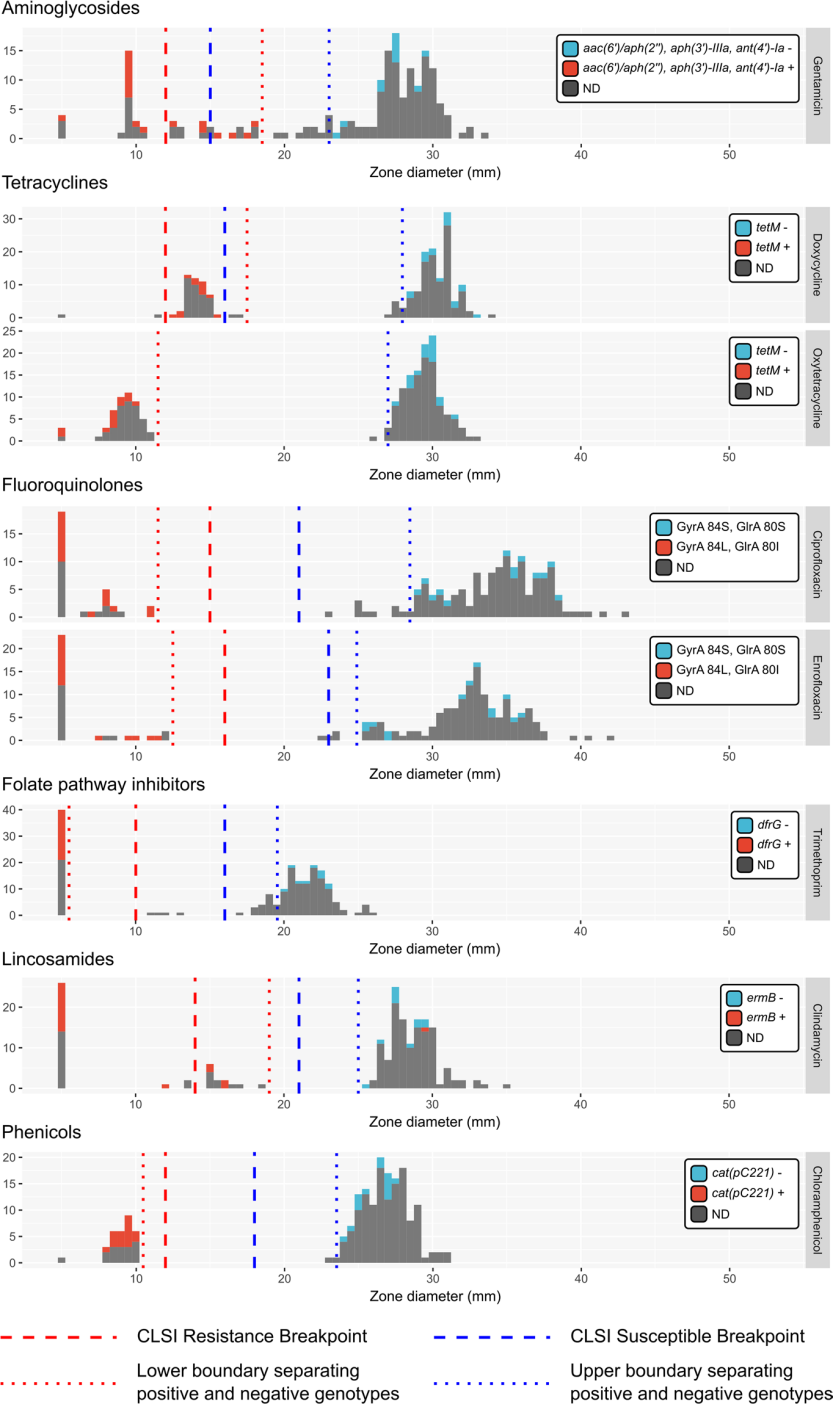
Non-beta-lactam antibiotic zone diameter measurements for all *S*. *pseudintermedius* isolates in this study. Histograms are presented for each antibiotic. A zone diameter of 5 mm is used to indicate a “no zone” result. Available genetic data are shown by the colour of each histogram, where antibiotic resistance gene positive genotypes are coloured light red, and negative genotypes are coloured light blue. Individual legends specify the relevant antibiotic resistance gene for each plot. Dotted lines specify the calculated optimal bounds for separating positive and negative genotypes in our data. Dashed lines show the R/I/S CLSI boundaries for antibiotics where breakpoint data were available at the time of publication.

Based on the obtained distribution of antibiotic resistance measurements, we randomly selected 29 MRSP and MDR-MRSP isolates for typing by whole genome sequencing (WGS). Illumina sequencing resulted in high-quality draft genome assemblies from all 29 isolates (Table S1). As was done for beta-lactam antibiotics, we examined the susceptibility profiles of each isolate and correlated zone diameter measurements with genotypes from sequenced genomes (Fig. 2 and Table 1).

Oxytetracycline and doxycycline were used to test for tetracycline-class resistance. Zone diameters for both these antibiotics showed a strong positive correlation (data not shown), and two distinct groups of zone diameter were obtained (Fig. 2). Tetracycline resistance is known to be conferred by the presence of the *tetM*, *tetL* and *tetK* genes in *S*. *pseudintermedius*^47,48^. Within isolates from our study, only *tetM* was identifiable in sequenced genomes, and we obtained a perfect correlation between genotype and phenotype between susceptible and intermediate classes. All *tetM*^+^ isolates fell in the intermediate category of resistance for doxycycline based on CLSI breakpoints.

Enrofloxacin and ciprofloxacin were used to test for fluoroquinolone-class resistance. Zone diameters for both enrofloxacin and ciprofloxacin showed a strong positive correlation (data not shown) and two distinct groups of zone diameter were obtained (Fig. 2). In *S*. *pseudintermedius*, fluoroquinolone resistance is thought to be associated with mutations at codon positions 84 and 80 in the *gyrA* and *glrA* genes, respectively^49^. Isolates from our study were either GyrA-84S:GlrA-80S or GyrA-84L:GlrA-80I. We obtained a perfect correlation between genotype and phenotype between susceptible and resistant classes, with GyrA-84L:GlrA-80I being the resistant genotype.

Similarly, trimethoprim and chloramphenicol showed a perfect correlation with the *dfrG* and *cat(pC221)* genotypes, respectively (Fig. 2). Intermediate levels of trimethoprim resistance have previously been seen in *S*. *aureus* with point mutations in *dfrA* and *dfrB* genes^50,51^. However, genomic data were not available to resolve the resistance phenotype of four isolates that showed intermediate levels of trimethoprim resistance.

Clindamycin resistance in *S*. *pseudintermedius* is conferred by the *ermB* gene and its expression can be constitutive or inducible^52^. Here, the relationship between the *ermB* genotype and constitutive resistance was imperfect. All isolates that were *ermB*^+^ and fell into either susceptible or intermediate categories for constitutive resistance also gave evidence of D-zone formation when tested for inducible resistance (Fig. 2 and Table 2); however, we noted that *S*. *pseudintermedius* generates relatively diffuse disk borders by this method, complicating interpretation of the assay.

**Table 2:** Resistance profiles of all isolates based on CLSI disk diffusion break points (VET08 4^th^ Edition and M100 27^th^ Edition) for *Staphylococcus pseudintermedius* (oxacillin and doxycycline) and *Staphylococcus* spp. (all other antibiotics). “ind = #” denotes the number of isolates in each category that gave evidence of an inducible clindamycin phenotype via D-formation in the erythromycin/clindamycin test.

Oxacillin	Doxycycline	Ciprofloxacin	Clindamycin	Trimethoprim	Chloramphenicol	Gentamicin	Resistance Profile	Count	Category	ST by WGS
R	I	R	I (ind = 2)	R	R	I	RIRIRRI	2	MDR-MRSP	496 (1)
R	I	R	I (ind = 2)	R	R	S	RIRIRRS	2	MDR-MRSP	496 (1)
R	I	R	R	R	R	S	RIRRRRS	6	MDR-MRSP	496 (3)
R	S	R	R	R	R	R	RSRRRRR	18	MDR-MRSP	71 (9)
R	S	R	S	R	R	R	RSRSRRR	2	MDR-MRSP	71 (1)
R	S	R	S	R	S	R	RSRSRSR	2	MDR-MRSP	71 (1)
R	R	S	S	R	S	S	RRSSRSS	1	MDR-MRSP	
R	I	S	I (ind = 3)	S	S	S	RISISSS	3	MRSP	84 (1)
R	I	S	R (ind = 3)	S	S	S	RISRSSS	3	MRSP	84 (1)
R	I	S	S	R	S	S	RISSRSS	3	MRSP	258 (1), 1173 (1)
R	I	S	S	S	S	S	RISSSSS	6	MRSP	1172 (1), 84 (1)
R	I	S	I (ind = 2)	R	S	S	RISIRSS	2	MRSP	277 (1)
R	S	S	I	S	S	R	RSSISSR	1	MRSP	
R	S	S	I (ind = 1)	S	S	S	RSSISSS	1	MRSP	
R	S	S	S	S	R	I	RSSSSRI	1	MRSP	
R	S	S	S	S	S	I	RSSSSSI	5	MRSP	64 (2)
R	S	S	S (ind = 1)	S	S	S	RSSSSSS	10	MRSP	1171 (1), 498 (1), 749 (2)
S	I	S	I	S	S	S	SISISSS	2	MSSP	
S	I	S	S	S	S	S	SISSSSS	18	MSSP	
S	R	S	S	S	S	S	SRSSSSS	1	MSSP	
S	S	S	R	S	S	S	SSSRSSS	2	MSSP	
S	S	S	S	I	S	S	SSSSISS	4	MSSP	
S	S	S	S	R	S	S	SSSSRSS	2	MSSP	
S	S	S	S	S	S	R	SSSSSSR	2	MSSP	
S	S	S	S	S	S	S	SSSSSSS	81	PSSP	

Sequence Type (ST), derived from alleles of seven MLST genes is given for isolates belonging to each resistance profile for which genomic data were available, the number of isolates sequenced is shown in parentheses.

Gentamicin zone diameters were relatively evenly distributed and did not fall into obvious groups. Gentamicin resistance is associated with the *aac(6*′*)/aph(2*″*)*:*aph(3*′*)-*IIIa:ant*(4*′*)-Ia* genotype in *S*. *pseudintermedius*. These genes are most frequently acquired together as part of a resistance cassette in staphylococci^53^. Several isolates that were defined as susceptible according to CLSI guidelines were observed to carry the *aac(6*′*)/aph(2*″*)*:*aph(3*′*)-*IIIa:ant*(4*′*)-Ia* genes, although the zone diameter measurements were capable of separating positive and negative genotypes at values slightly higher than those of the CLSI standards (Fig. 2).

Full resistance profiles were defined for seven classes of antibiotic using available CLSI breakpoints (Table 2). Pan-susceptibility was the most commonly identified resistance profile. MRSP isolates possessed between 0 and 6 classes of resistance in addition to *mecA*-mediated broad-spectrum beta lactam resistance (including intermediate resistance). All MDR isolates (possessing three or more classes of antibiotic resistance) were MDR-MRSP; MDR-MSSP has been reported elsewhere^10^ but was not identified in this study.

Seven-gene MLST locus data were extracted from each sequenced genome in order to assign a sequence-type (ST) to each isolate. A total of 11 different STs were identified among the 29 sequenced isolates; STs 64, 71, 84, 258, 277, 496, 498 and 749 as well as three previously undefined STs 1171, 1172 and 1173 (Table 2). The most highly drug resistant variants that we identified all belonged to either ST 71 or ST 496.

### MALDI-TOF spectra distinguish between some common sequence types of *S*. *pseudintermedius*

MALDI-TOF spectroscopy has revolutionised veterinary and medical diagnostics, making rapid species identification robust, simple and affordable^54^. Having demonstrated that *S*. *pseudintermedius* species detection by MALDI-TOF is both sensitive and specific for isolates in our study, we tested its ability to distinguish identified subtypes of *S*. *pseudintermedius*.

**Figure 3:**
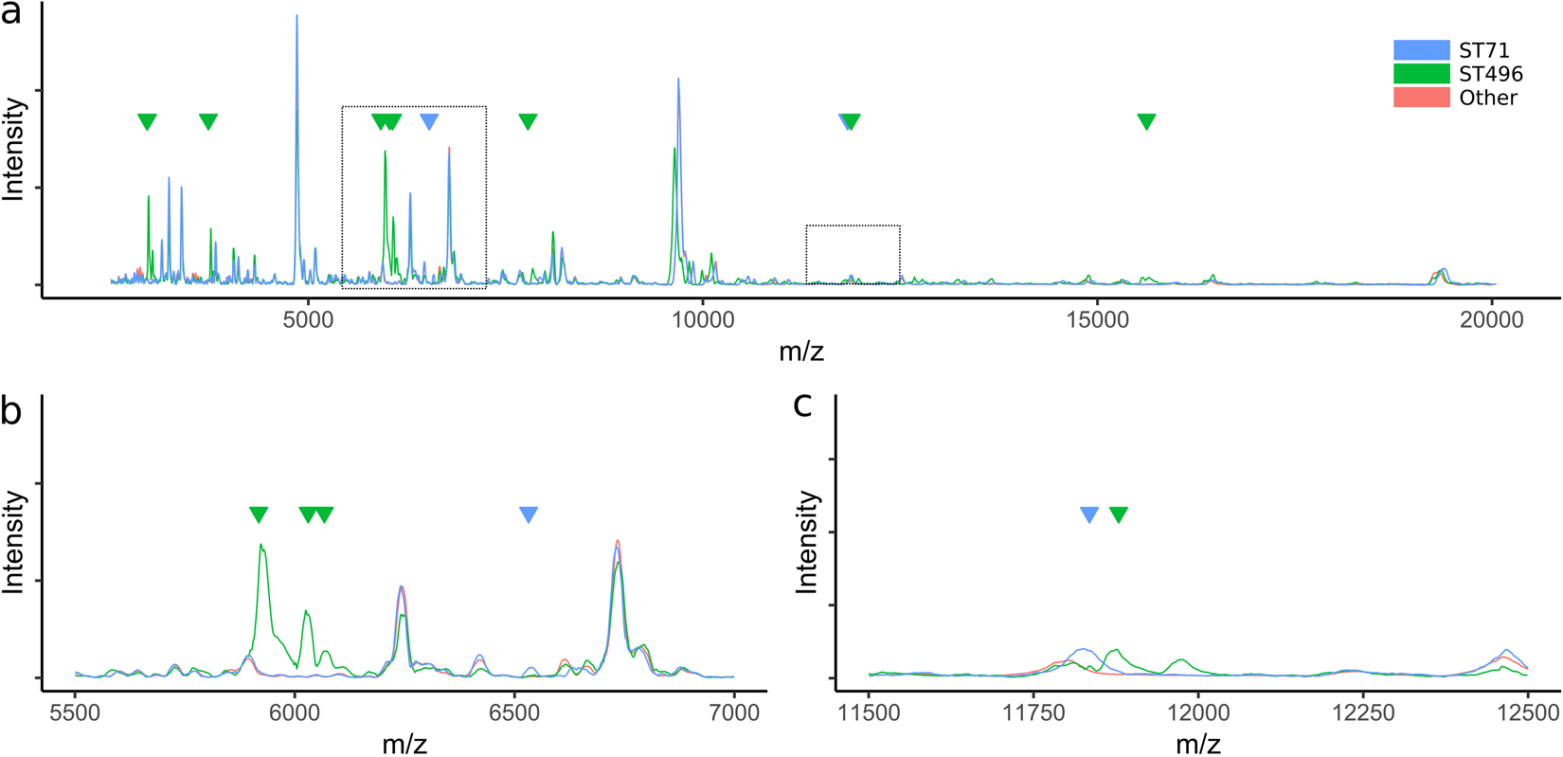
MALDI-TOF spectra of *S*. *pseudintermedius* belonging to ST 71 and ST 496 compared to other *S*. *pseudintermedius* isolates. Discriminatory peaks are indicated with arrows; two distinct peaks were present in ST 71 spectra and multiple distinct peaks in ST 496 spectra. Zoomed-in views of the highlighted areas are presented in panels b and c.

MRSP was indistinguishable from MSSP by either variance-based methods (Fig. S2) or peak-specific discriminant analysis (data not shown). However, 98% and 99% accuracy was achieved when discriminating ST 71 isolates and ST 496 isolates, respectively. Major variations at multiple peak positions were visible for all ST 496 isolates, which were specific markers of this group (Figs 3 and S3). ST 71 isolates showed much more subtle variation in their spectra; characteristic low-level peaks were observed at m/z values of 6543 and 11799. The latter of these appeared to be the result of a slight peak-shift, with all other isolates demonstrating a similar intensity peak at m/z 11770 (Fig. 3c).

### WGS predicts infrequent, ancestral SCC*mec* acquisition, followed by subsequent global dispersal

Understanding the dynamics of antibiotic resistance acquisition is important when developing antibiotic stewardship or biosecurity strategies. The availability of genomic data for *S*. *pseudintermedius* is rapidly increasing (eg.^55–58^) but, to date, population structure estimates and genealogies for this species have principally been constructed using seven-gene MLST data^10,59^. To test how genomic data influenced estimates of resistance gene acquisition frequency, we constructed phylogenies from a comprehensive collection of global MLST profiles and WGS data for *S*. *pseudintermedius*. At the time of analysis, a total of 949 different seven-gene MLST definitions were identified by combining PubMLST and genomic data. The 223 published genome sequences came from a subset of 50 unique MLST-types. With the exception of some datasets from the UK, the vast majority of published genomic data come from MRSP variants.

Our S. *pseudintermedius* genealogy reconstructions using seven-gene MLST loci produced a star-like phylogeny with poor support (Fig. S4). This has been observed previously, and the lack of phylogenetic resolution could be explained by either insufficient variant sites or high rates of recombination at these loci^60^.

**Figure 4:**
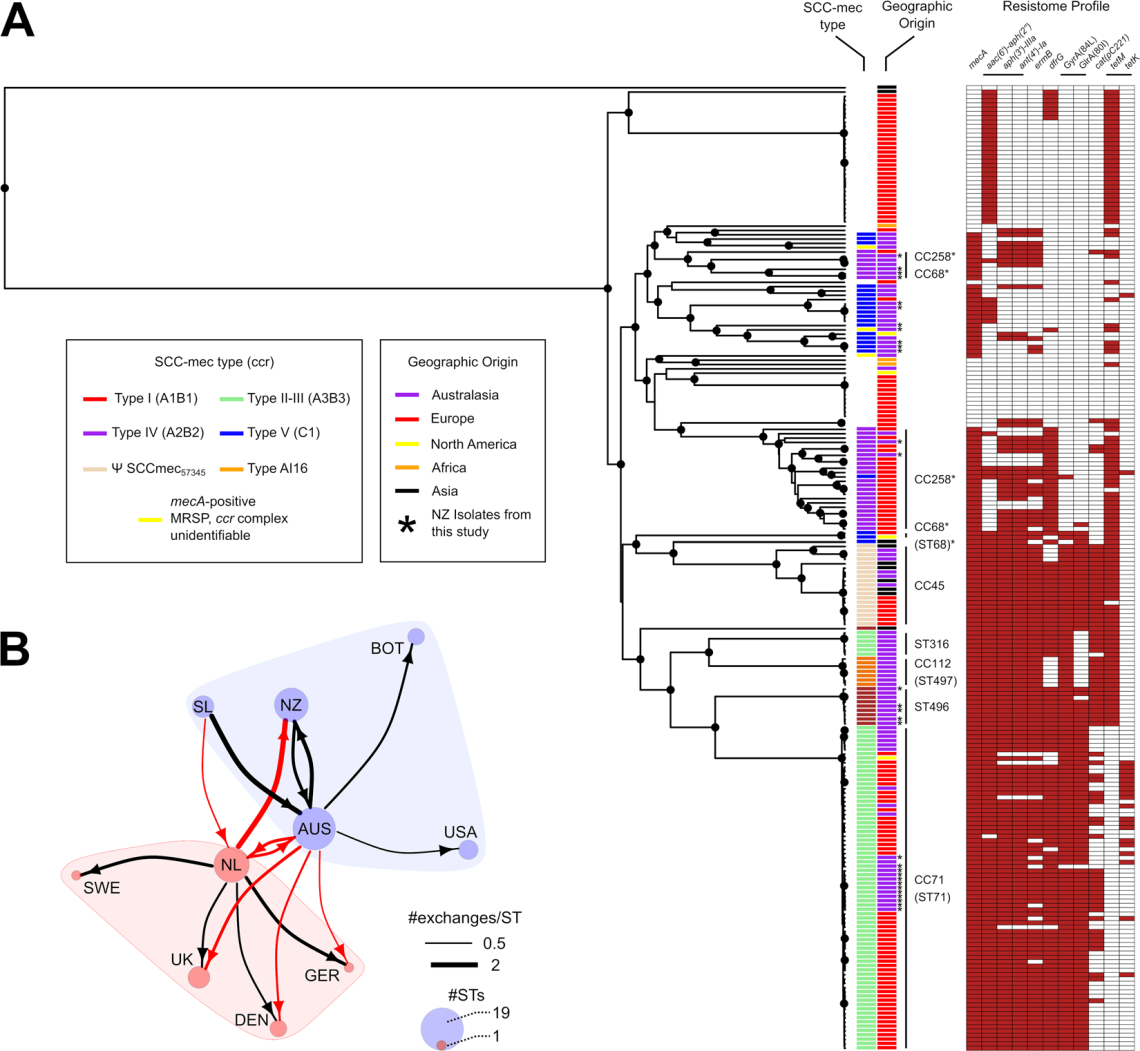
Comparative genomics analysis of global MRSP. (**A**) Predicted phylogenetic relationship between 223 genomes. Dendrogram nodes with posterior probabilities of 1 are indicated by circles. Stars denote isolates generated as part of this study. Resistome profiles are displayed as a heatmap indicating presence (red) or absence (white) of the genotype indicated at the head of each column. (**B**) Summary of global exchange predicted from the presented phylogeny. Network arrow thickness denotes an estimate of the frequency of exchange between each country in the analysis, and is corrected for reported sequence-type diversity from each location. Node size indicates the number of different MLST types of *S*. *pseudintermedius* that are present in published genomes from each location. Coloured boundaries represent discriminatory groupings defined by community analysis of the undirected network. Country abbreviations used are; UK: United Kingdom, SWE: Sweden, DEN: Denmark, GER: Germany, NL: Netherlands, AUS: Australia, NZ: New Zealand, SL: Sri Lanka, BOT: Botswana, USA: United States of America.

Contrastingly, whole genome phylogenetic analysis generated a well-supported bifurcating topology for the subset of available strain types (Fig. 4A). Members of MLST-based clonal complexes (CCs) 71, 112 and 45 were identifiable as monophyletic clades in this analysis, whereas members of clonal complexes 68 and 258 formed paraphyletic groups that clustered loosely by resistance gene profile. Resistance gene profiles showed that multidrug resistance genotypes to numerous classes of antibiotics were most strongly associated with CC 45, CC 112, CC 71, ST 316 and ST 496. TetK-based tetracycline resistance was common but not conserved in members of ST71, whereas TetM-based tetracycline resistance was more common in other lineages. Ciprofloxacin resistance-associated mutations GyrA(84 L) and GlrA(80I) were most frequently present together, with the exception of ST 316 and ST 497 which possessed only the GyrA(84 L) mutation.

MRSP variants formed mainly monophyletic groups which clustered by SCC*mec* type. Despite the over-representation of MRSP strains in the global genomic data set, parsimony-based ancestral reconstructions of each lineage’s SCC*mec* state identified a methicillin susceptible state at the root of the phylogeny (Fig. S5). Furthermore, MRSP diversity associated with all *S*. *pseudintermedius* strains was estimated to have derived from as few as 14 independent acquisitions of different SCC*mec* cassettes (Table S2). These analyses also predicted infrequent loss of the SCC*mec* cassette, however, loss events were associated with nodes that had lower posterior support in the phylogeny. Most monophyletic SCC*mec* clades consisted of isolates derived from multiple countries across the world, showing that stable methicillin-resistant lineages are likely to have circulated the world on multiple occasions.

Community analysis of geographic exchange estimates based on similar ancestral state reconstructions, and corrected for reported ST diversity per country, allowed us to estimate the frequency of exchange of *S*. *pseudintermedius* lineages between countries. Significant inter-connectivity was observed between *S*. *pseudintermedius* lineages in European countries, as well as frequent bidirectional exchanges between New Zealand and Australia, and Australia and the Netherlands (Fig. 4B). However, it should be noted that sample diversity was heavily biased towards isolates of European and Australasian origin.

**Figure 5:**
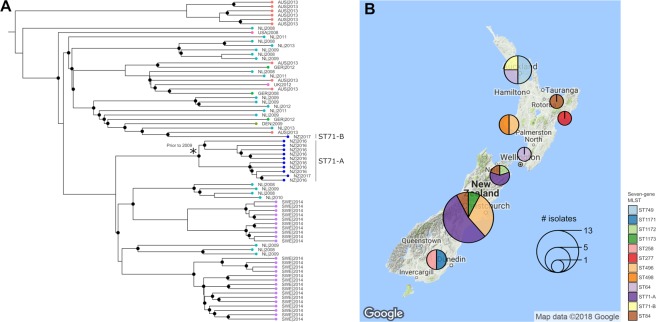
Comparative genomics analysis of ST 71. (**A**) Predicted phylogenetic relationship between genomes belonging to MLST type 71. Dendrogram nodes with posterior probabilities greater than 0.9 are indicated by circles. Leaf tips are coloured by geographical origin. Distinct monophyletic lineages of New Zealand isolates “ST 71-A” and “ST 71-B” are highlighted. The star highlights the latest predicted date of emergence of ST 71-A, corresponding to the upper range of the posterior distribution of predicted date. All other dating information is left off due to lack of support. (**B**) The geographical distribution of distinct lineages of MRSP types that has been reported from New Zealand to date.

The frequent global exchange of *S*. *pseudintermedius* lineages was most evident in highly sampled lineage ST 71. We identified two independent New Zealand lineages of ST 71 in these data: ST 71-A (n = 10) and ST 71-B (n = 1) (Fig. 5A). ST 71-A formed a monophyletic, New Zealand-specific group indicative of endemic circulation following a single introduction of a common ancestral strain. Divergence estimates for lineage ST 71-A suggested that the latest possible date for this common ancestor was around 2009, five years prior to the first reported observation of MRSP in New Zealand^16^. However, date parameter estimates showed very broad posterior distributions (median = 1977, 95% HPD = 1944–2004) thus precise estimates of the dates of introduction of the different lineages were not possible. Examination of the geographical distribution of identified MRSP lineages from across New Zealand showed that ST 71-A had only been reported in the South Island while ST 84 and ST 496 had been reported on multiple occasions in distinct geographical settings in both the North and South islands, suggesting multiple lineages may be endemic in the country (Fig. 5B). ST 71-B appeared to be recently introduced from Australia and only reported in the North Island.

## Discussion

MRSP is becoming a major pathogen in veterinary hospitals across the world and is difficult to treat as many MRSP isolates are also MDR^61^. Global studies of MRSP carriage in healthy animals report highly varied observations, ranging from approximately 2% to 50% prevalence although surveillance data is limited^62,63^.

In this study, we performed a multifaceted analysis of New Zealand *S*. *pseudintermedius* isolates to examine how technology may be used to improve our understanding and surveillance practices surrounding MRSP. MALDI-TOF identification is a valuable tool for diagnostic laboratories, however there are mixed reports in the literature about the specificity of MALDI-TOF typing for the identification of *S*. *pseudintermedius*^20–22^. Our work on New Zealand *S*. *pseudintermedius* isolates from dogs demonstrates both sensitive and specific species-level identification of *S*. *pseudintermedius* by Bruker MALDI-TOF MS, most likely corresponding to improvements in the Bruker reference database. Additionally, we used PCR and genomics to correlate resistance genotypes to antibiotic susceptibilities as measured by disk diffusion. For isolates in our dataset, we found that oxacillin, cephalexin, cefpodoxime and cefovecin could be used as reliable indicators of the *mecA* genotype while ampicillin, amoxycillin and amoxycillin/clavulanate were poor indicators due to the existence of non-*mecA*-mediated resistance mechanisms. Many of these mechanisms have been previously described for ampicillin resistance^45^; however, we identified a cluster of *S*. *pseudintermedius* isolates that demonstrated an unknown mechanism resulting in increased resistance to amoxycillin-clavulanate with no increased resistance to similar concentrations of amoxycillin. We found that existing CLSI breakpoints classified many *mecA*^+^ isolates as having intermediate levels of resistance to both cefpodoxime and cefovecin, and noted a single observation of a likely misclassification where an isolate possessing *mecA* was identified as susceptible to cefovecin. For gentamicin, we also observed many genotype positive isolates of *S*. *pseudintermedius* that were classified as susceptible when using CLSI breakpoints established for “*Staphylococcus* spp.”. Together, these observations suggest that *S*. *pseudintermedius* may require further establishment of species-specific break points for disk diffusion experiments to many antibiotics. This is perhaps unsurprising, as reference standards are still evolving for *S*. *pseudintermedius* due to its relatively recent reclassification^4^.

We performed in-depth analysis of the MALDI-TOF spectral data from more than 100 *S*. *pseudintermedius* isolates and coupled this with WGS analysis where possible. Within our sample set, MALDI-TOF MS could be used to discriminate between sequence types ST 71 and ST 496. This level of categorical precision is comparable to that of single-locus or multi-locus sequence typing, but can be achieved more rapidly and at much lower cost. Although our analysis was constrained to strain variants that we could isolate in New Zealand at the time of this study (and therefore the number of isolates tested was low), our observations highlight a valuable use of MALDI-TOF MS in the surveillance of *S*. *pseudintermedius* at species and genetic variant level. However, it would be necessary to validate such discriminatory analyses in local settings against up-to-date panels of *S*. *pseudintermedius* isolates belonging to all regionally circulating lineages.

One of the prerequisites to successful and actionable surveillance is precise typing. At the individual level, standardised strain identification aids our ability to treat infection by drawing on prior stain-specific examples. At the population level, understanding the incidence of circulating strains and their epidemiology allows biosecurity measures to be adapted in the face of changing infection landscapes. While MLST-based schemes have been used to establish useful classifications for *S*. *pseudintermedius*, our analyses suggest that increasingly recognised species diversity and the lack of phylogenetic resolution provided by such loci does not allow for accurate definitions of population structure. This ultimately limits our understanding of the origins of different lineages and the evolution of antibiotic resistance in *S*. *pseudintermedius*. WGS offers an improved method for addressing such questions, and here we used available global data to examine the frequency of historical SCC*mec* acquisition, demonstrating that the main circulating lineages acquired resistance ancestrally on as few as 14 occasions. This estimate is greatly reduced compared to that derived from 7-gene MLST data^60^ and is more comparable with similar estimates in *S*. *aureus*^64^, challenging the assumption that antibiotic resistance acquisition by horizontal gene transfer is commonplace for this species (at least for SCC*mec*). However, the observed reduction is due to both resolved phylogenetic relationships between lineages and the availability of data from only a subset of STs. Global reports suggest that these resistant strain variants are becoming increasingly prevalent worldwide^60^. However, there is geographic bias and a dearth of MSSP in available WGS data. Coupled with the demonstrated imprecisions of MLST phylogeny, this currently makes it unclear which lineages are susceptible precursors to many of the dominant MRSP lineages and whether or not they are still in circulation. This, in turn, complicates the evaluation of selection pressures that are likely driving the worldwide increase in MRSP and MDR-MRSP observations.

Our genomic analyses of MRSP isolates suggest that multiple types (sequence types 64, 71, 84, 258, 277, 496, 498, 749 1171, 1172 and 1173) have been imported into New Zealand and that some infections are likely to be the result of autochthonous transmission. In this and other settings where MRSP is an established endemic pathogen, unbiased population-level evaluations of prevalence will be required to assess the scale of the burden to public (and veterinary) health. Antibiotic treatments that precede microbiological diagnostics commonly increase the relative abundance of on-animal MRSP, which in turn may lead to increased population-level MRSP prevalence^59^. This problem is amplified when strains with multiple resistance phenotypes become prevalent as the use of single antibiotics or antimicrobials co-selects for multiple classes of resistance^65^. Faced with such a challenge to public health, an important step to combat MRSP and similar healthcare-associated MDR pathogens is real-time surveillance and the use of precise diagnostic techniques such as MALDI-TOF MS and WGS in a context that can be linked to clinical and epidemiological data^15^. Standardised surveillance and reporting can effectively inform antibiotic stewardship programmes, infection control strategies, interventions and help to stabilise or reduce antibiotic resistance prevalence^66^.

## Supplementary information

Supplementary Information

## Data Availability

Assembled draft genomes were deposited to NCBI under BioProject number PRJNA473042.
