# Supplementary material for: Combining MALDI-TOF and genomics in the study of methicillin resistant and multidrug resistant Staphylococcus pseudintermedius in New Zealand
Source: Sci Rep. 2019 Feb 4;9:1271. doi: 10.1038/s41598-018-37503-9 (PMC6361924; doi:10.1038/s41598-018-37503-9)
Supplement: Supplementary file 1 — Supplementary Information [file 41598_2018_37503_MOESM1_ESM.docx]

**SUPPLEMENTARY INFORMATION**

**Combining MALDI-TOF and genomics in the study of methicillin resistant and multidrug resistant *Staphylococcus pseudintermedius* in New Zealand**.

Shahista Nisa^a^, Clément Bercker^a,b^, Anne C Midwinter^a^, Ian Bruce^c^, Chris F. Graham^d^, Pierre Venter^e^, Allan Bell^f^, Nigel P. French^g^, Jackie Benschop^a^, Karen M. Bailey^d^, David A. Wilkinson*^a,g^

^a^Molecular Epidemiology and Public Health Laboratory, Hopkirk Research Institute, Massey University, Palmerston North, New Zealand;

^b^Ecole Nationale Veterinaire de Toulouse, Toulouse, France;

^c^NZVP (IDEXX), Palmerston North, New Zealand;

^d^Gribbles Veterinary, Christchurch, New Zealand;

^e^Fonterra Research & Development Centre, Palmerston North, New Zealand;

^f^Dermvetonline, Auckland, New Zealand;

^g^New Zealand Food Safety Science and Research Centre, Massey University, Palmerston North, New Zealand.

Supplementary Figures and Tables

**
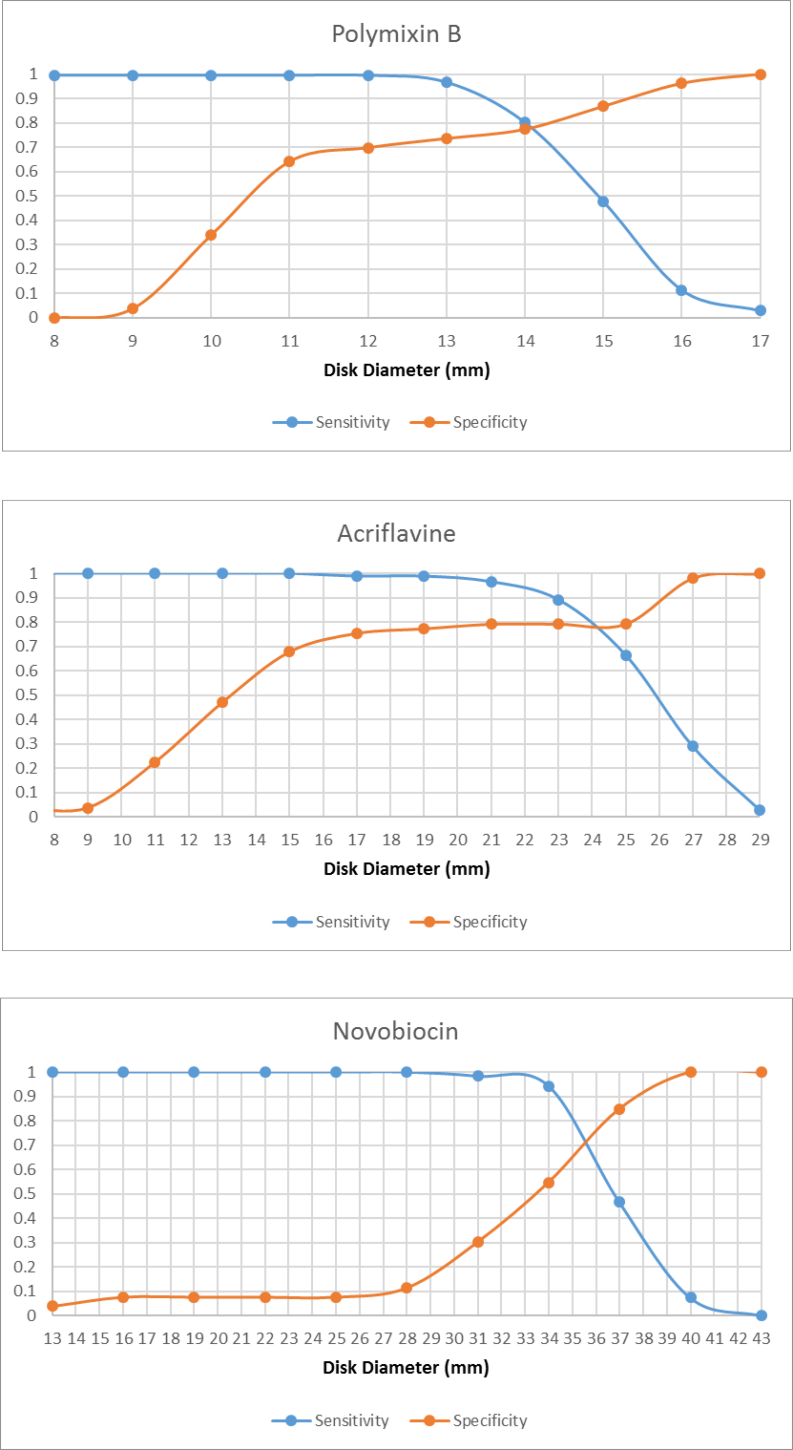
**

**Figure S1: Sensitivity and specificity calculations for species prediction using Polymixin B, Acriflavine and Novobiocin zone diameters.** Numbers are calculated across all zone diameter thresholds (x-axis), defining isolates with zone diameters greater than each threshold as “*S. pseudintermedius*”, and isolates with disk diameters lower than the threshold as “not *S. pseudintermedius*”. Sensitivity and specificity are calculated relative to the MALDI-TOF MS species call of each isolate, which we assume to be the ground truth in this example.

**Figure S2:**

**
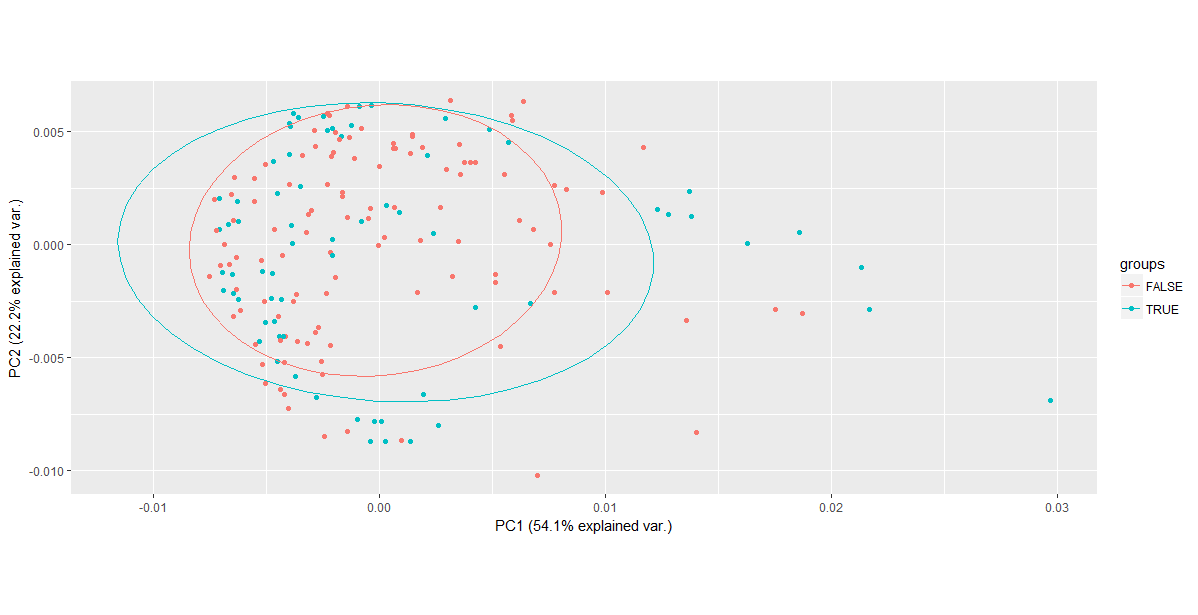
**

**Figure S2: Principal component analysis of MALDI-TOF spectra.** Data are plotted in principal components 1 and 2, accounting for 76.3% of the total variance in the spectral data. Colours represent MRSP isolates (red) and MSSP isolates (blue).

**Figure S3:**

**
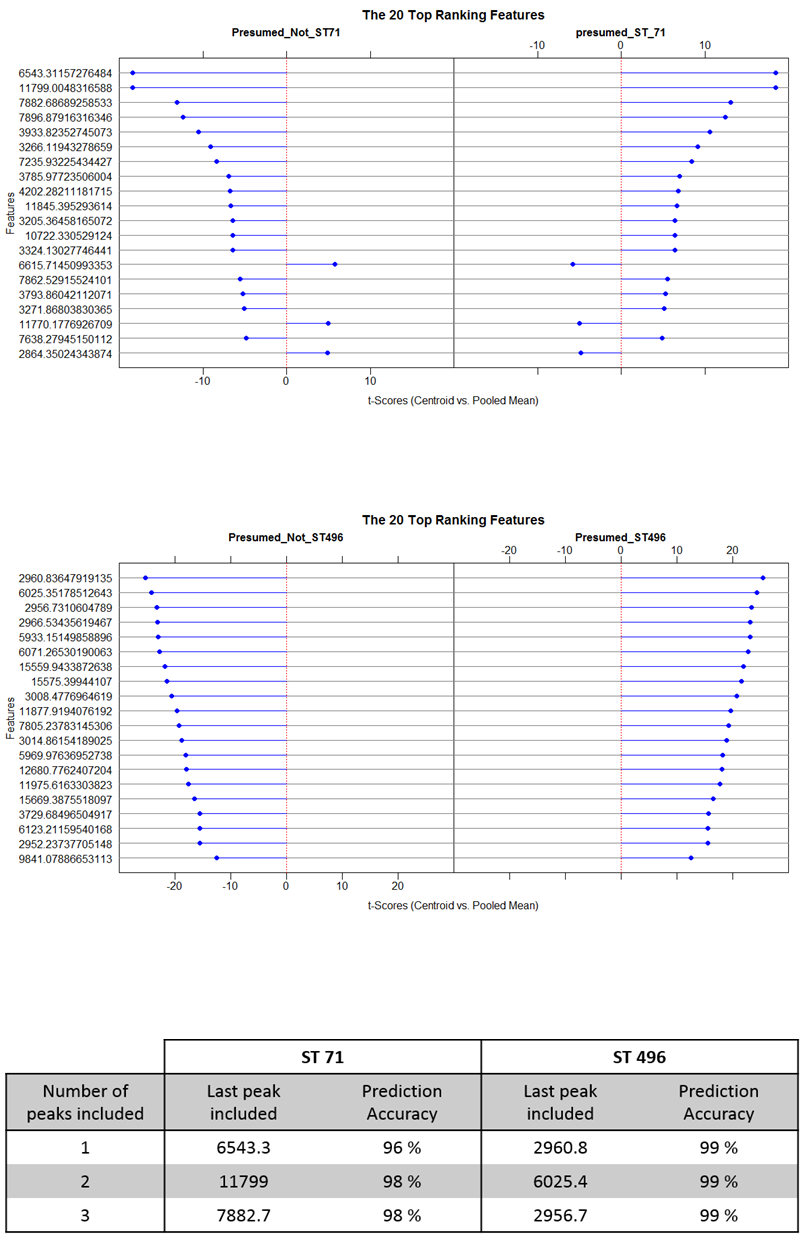
**

**Figure S3:** Discriminant analysis of MALDI-TOF MS peaks of ST71 and ST 496.

**Figure S4:**

**
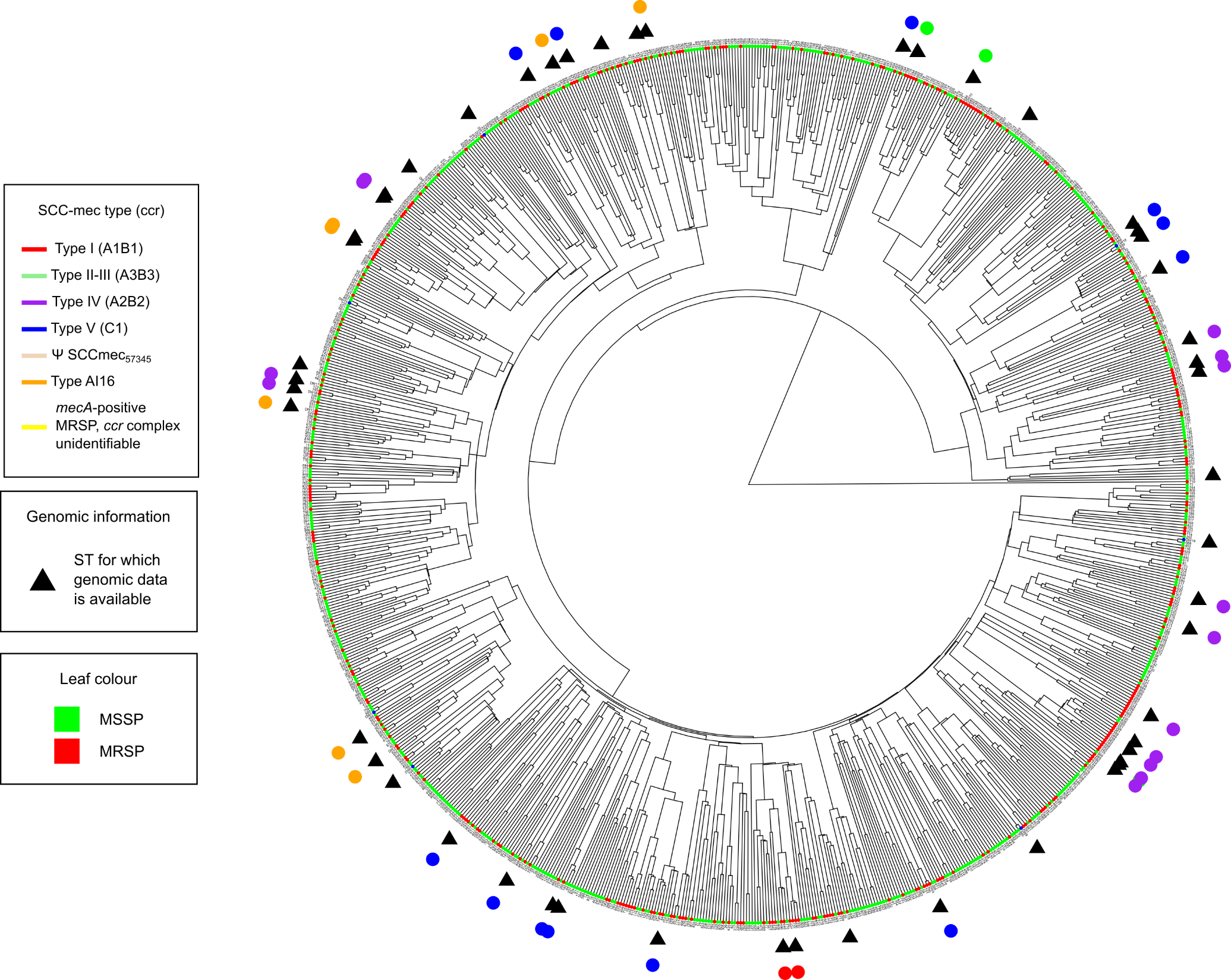
**

**Figure S4:** Phylogenetic analysis of 949 seven-gene MLST profiles identified from PubMLST ^24^.


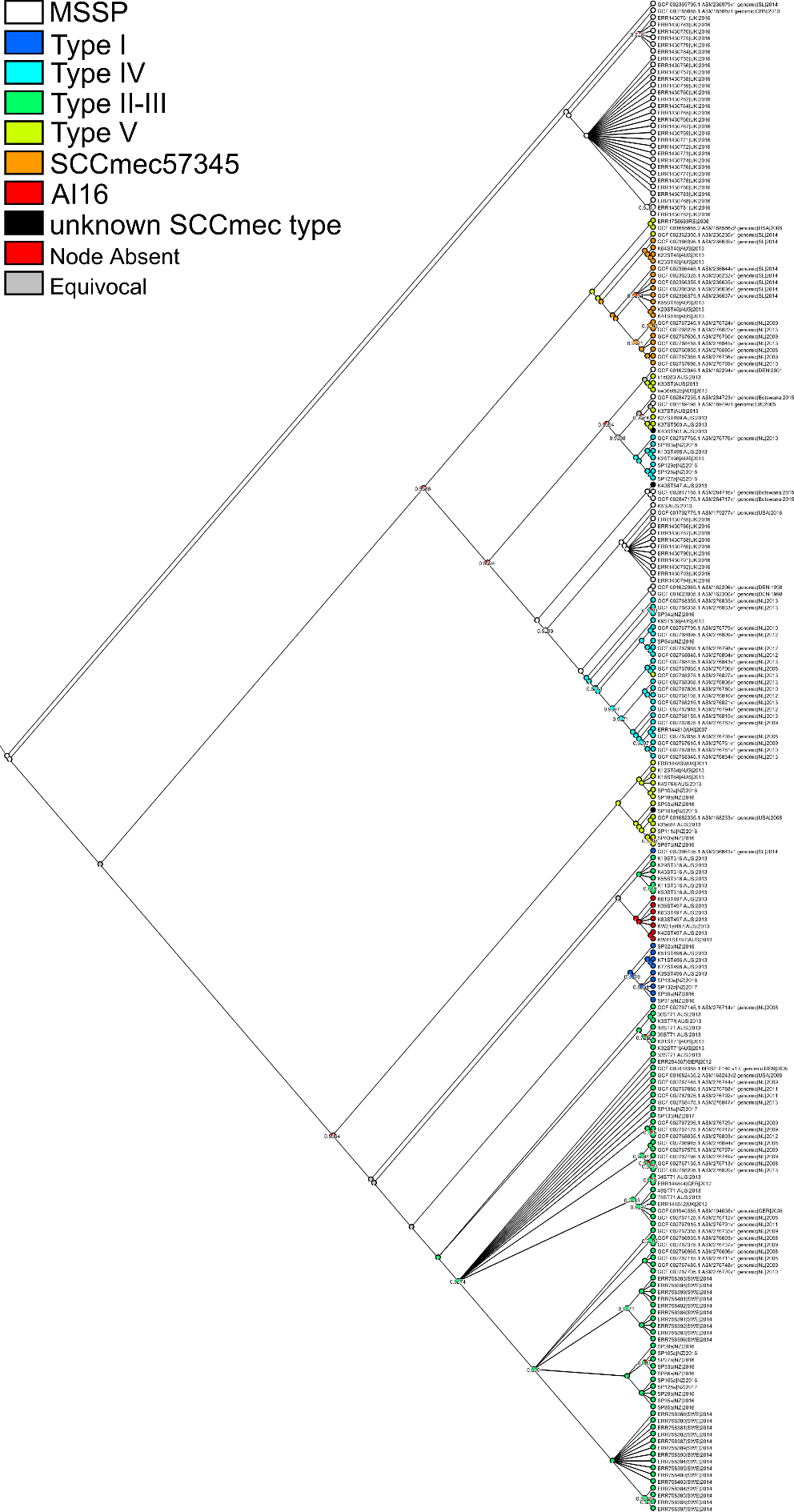


**Figure S5:** Mesquite ancestral states reconstruction of SCC*mec* inheritence. Node colours indicate most likely ancestral state based on parsimony.

| **Isolate ID** | **Host** | **Year** | **# Contigs (>200 bp)** | **Genome size (bp)** | **N50** | **Accession #** |
| --- | --- | --- | --- | --- | --- | --- |
| SP102a | *Canis lupus familiaris* | 2016 | 74 | 2,641,377 | 123,752 | QHIL00000000 |
| SP103a | *Canis lupus familiaris* | 2016 | 97 | 2,655,168 | 141,220 | QHIK00000000 |
| SP105a | *Canis lupus familiaris* | 2016 | 74 | 2,765,564 | 151,240 | QHIJ00000000 |
| SP106a | *Canis lupus familiaris* | 2016 | 67 | 2,764,860 | 151,435 | QHII00000000 |
| SP111a | *Canis lupus familiaris* | 2016 | 78 | 2,647,151 | 140,610 | QHIH00000000 |
| SP126a | *Canis lupus familiaris* | 2016 | 90 | 2,638,083 | 161,618 | QHIG00000000 |
| SP127a | *Canis lupus familiaris* | 2016 | 82 | 2,625,558 | 145,481 | QHIF00000000 |
| SP128a | *Canis lupus familiaris* | 2017 | 73 | 2,764,440 | 145,551 | QHIE00000000 |
| SP129a | *Canis lupus familiaris* | 2016 | 82 | 2,538,059 | 160,801 | QHID00000000 |
| SP130a | *Canis lupus familiaris* | 2016 | 84 | 2,804,634 | 123,580 | QHIC00000000 |
| SP131a | *Canis lupus familiaris* | 2017 | 66 | 2,787,598 | 158,635 | QHIB00000000 |
| SP132a | *Canis lupus familiaris* | 2017 | 82 | 2,753,584 | 123,580 | QHIA00000000 |
| SP18a | *Canis lupus familiaris* | 2016 | 82 | 2,640,597 | 123,752 | QHJC00000000 |
| SP27a | *Canis lupus familiaris* | 2015 | 63 | 2,801,487 | 158,662 | QHJB00000000 |
| SP28a | *Canis lupus familiaris* | 2014 | 63 | 2,761,781 | 150,786 | QHJA00000000 |
| SP29a | *Canis lupus familiaris* | 2015 | 89 | 2,772,374 | 143,042 | QHIZ00000000 |
| SP30a | *Canis lupus familiaris* | 2015 | 98 | 2,744,803 | 63,102 | QHIY00000000 |
| SP31a | *Canis lupus familiaris* | 2016 | 80 | 2,752,500 | 133,591 | QHIX00000000 |
| SP32b | *Canis lupus familiaris* | 2015 | 104 | 2,696,991 | 123,580 | QHIW00000000 |
| SP33a | *Canis lupus familiaris* | 2015 | 138 | 2,832,101 | 238,846 | QHIV00000000 |
| SP34a | *Canis lupus familiaris* | 2014 | 83 | 2,728,216 | 128,642 | QHIU00000000 |
| SP35a | *Canis lupus familiaris* | 2015 | 77 | 2,809,361 | 143,269 | QHIT00000000 |
| SP53a | *Canis lupus familiaris* | 2016 | 69 | 2,685,799 | 115,125 | QHIS00000000 |
| SP83a | *Canis lupus familiaris* | 2016 | 232 | 2,716,058 | 140,960 | QHIR00000000 |
| SP84a | *Canis lupus familiaris* | 2016 | 80 | 2,730,905 | 130,580 | QHIQ00000000 |
| SP85a | *Canis lupus familiaris* | 2016 | 72 | 2,771,887 | 143,042 | QHIP00000000 |
| SP86a | *Canis lupus familiaris* | 2016 | 73 | 2,805,780 | 238,846 | QHIO00000000 |
| SP87a | *Canis lupus familiaris* | 2016 | 69 | 2,643,760 | 140,652 | QHIN00000000 |

**Supplementary Table S1**: Illumina sequencing assembly statistics and metadata of 29 sequenced MRSP and MDR-MRSP isolates

| Ancestral state change | Number of predicted events (averaged across all phylogenetic trees) |
| --- | --- |
| **MSSP to MRSP** | **6.7** |
| **MRSP_a_ to MRSP_b_** | **7.8** |
| **MRSP to MSSP** | **1.4** |

**Supplementary Table S2:** Enumeration of ancestral state predictions in changes of SCC*mec* cassette types.
